# Selective dissociation between LSD1 and GFI1B by a LSD1 inhibitor NCD38 induces the activation of *ERG* super-enhancer in erythroleukemia cells

**DOI:** 10.18632/oncotarget.24774

**Published:** 2018-04-20

**Authors:** Ryusuke Yamamoto, Masahiro Kawahara, Shinji Ito, Junko Satoh, Goichi Tatsumi, Masakatsu Hishizawa, Takayoshi Suzuki, Akira Andoh

**Affiliations:** ^1^ Department of Hematology and Oncology, Graduate School of Medicine, Kyoto University, Kyoto, Kyoto, Japan; ^2^ Department of Medicine, Shiga University of Medical Science, Otsu, Shiga, Japan; ^3^ Medical Research Support Center, Graduate School of Medicine, Kyoto University, Kyoto, Kyoto, Japan; ^4^ Department of Chemistry, Graduate School of Medical Science, Kyoto Prefectural University of Medicine, Kyoto, Kyoto, Japan; ^5^ CREST, Japan Science and Technology Agency (JST), Kawaguchi, Saitama, Japan

**Keywords:** LSD1, super-enhancer, GFI1B, RUNX1, leukemia

## Abstract

Lysine-specific demethylase 1 (LSD1) is a histone modifier for transcriptional repression involved in the regulation of hematopoiesis. We previously reported that a LSD1 inhibitor NCD38 induces transdifferentiation from erythroid lineage to granulomonocytic lineage and exerts anti-leukemia effect through de-repression of the specific super-enhancers of hematopoietic regulators including *ERG* in a human erythroleukemia cell line, HEL. However, the mechanistic basis for this specificity of NCD38 has remained unclear. Herein, we report major partners associated with LSD1 and clarify the mechanism in HEL cells. Proteome analysis identified 54 candidate proteins associated with LSD1, including several transcription factors such as GFI1B and RUNX1 as well as BRAF-histone deacetylase complex (BHC) components such as CoREST, HDAC1, and HDAC2. NCD38 selectively disrupted the interaction of LSD1 with GFI1B but not with RUNX1, CoREST, HDAC1 and HDAC2. *Erg* was downregulated in murine erythroid progenitors with prominent upregulation of *Gfi1b*. NCD38 induced ERG and attenuated an erythroid marker CD235a in HEL while this attenuation was mimicked by the lentiviral overexpression of ERG. The *ERG* super-enhancer contained the conserved binding motif of GFI1B and was actually occupied by GFI1B. NCD38 dissociated LSD1 and CoREST but not GFI1B from the *ERG* super-enhancer. Collectively, the selective separation of LSD1 from GFI1B by NCD38 restores the *ERG* super-enhancer activation and consequently upregulates ERG expression, inducing the transdifferentiation linked to the anti-leukemia effect.

## INTRODUCTION

Transcription factors (TFs) play essential roles in the regulation of normal hematopoiesis [1]. Abnormalities and dysregulation of TFs are major causes of leukemogenesis [2–4]. TFs cooperatively or antagonistically regulate each other within transcriptional networks [5], and this regulation is controlled via the activation of enhancers near or inside the gene bodies of TFs [6]. As enhancers contain multiple binding motifs of TFs, they are variously activated depending on TFs recruitment. In particular, super-enhancers (SEs) which are concatenated enhancers confer higher transcriptional activity than typical enhancers [7]. Activated SEs are found near the genes that define cell identity in embryonic stem cells and oncogenes in tumor cells, indicating that the precise control of SEs is essential in normal cell differentiation and dysregulation of SEs links to tumor pathogenesis [8]. SEs are occupied not only by TFs but also by mediators, chromatin regulators and transcription apparatus, and activated in a lineage specific manner [7]. On the other hand, the proper shut-down of SEs that are no longer required during the process of differentiation is also essential to secure the lineage specificity, however, less is known about the detailed mechanism.

Lysine-specific demethylase 1 (LSD1, also known as KDM1A, BHC110, AOF2, or KIAA0601), which was identified as a histone demethylase and a transcriptional corepressor [9], possesses a flavin containing amine oxidase that catalyzes mono- and di-methylated histone 3 lysine 4 (H3K4me1 and H3K4me2). LSD1 also interacts with RE1 silencing transcription factor corepressor (CoREST, also known as RCOR1) and histone deacetylase 1 and 2 (HDAC1 and HDAC2, respectively) [10, 11]. LSD1 is therefore involved in deacetylation of acetylated histone 3 lysine 27 (H3K27ac). Histone modifications are a crucial manner to regulate gene transcriptions [12]. In particular, as H3K4me1 and H3K27ac are general markers of poised or activated enhancers [13], LSD1 is supposed to function as a repressor of enhancers. In fact, loss of Lsd1 causes pancytopenia and is associated with increased levels of H3K27ac on the enhancers of Lsd1 target genes in murine hematopoietic-lineage cells [14].

We recently reported that a novel LSD1 inhibitor, NCD38, can induce myeloid differentiation in human erythroleukemia (HEL) cells and activate approximately 500 SEs [15]. According to the rank order SE (ROSE) analysis, the SE of *Ets related gene (ERG)*, one of the key TFs for hematopoiesis [5], was at the highest rank among them. These data indicate that LSD1 is involved in blood cell differentiation through repressing the SEs of the key hematopoietic TFs, however, it has remained unclear how LSD1 preferentially regulates such SEs. In this study, we attempt to identify binding partners for LSD1 in HEL cells, and to clarify the mechanism that NCD38 can selectively de-repress particular SEs with these partners, focusing on the *ERG*-SE.

## RESULTS

### Identification of LSD1 partners in HEL cells

**Figure 1:**
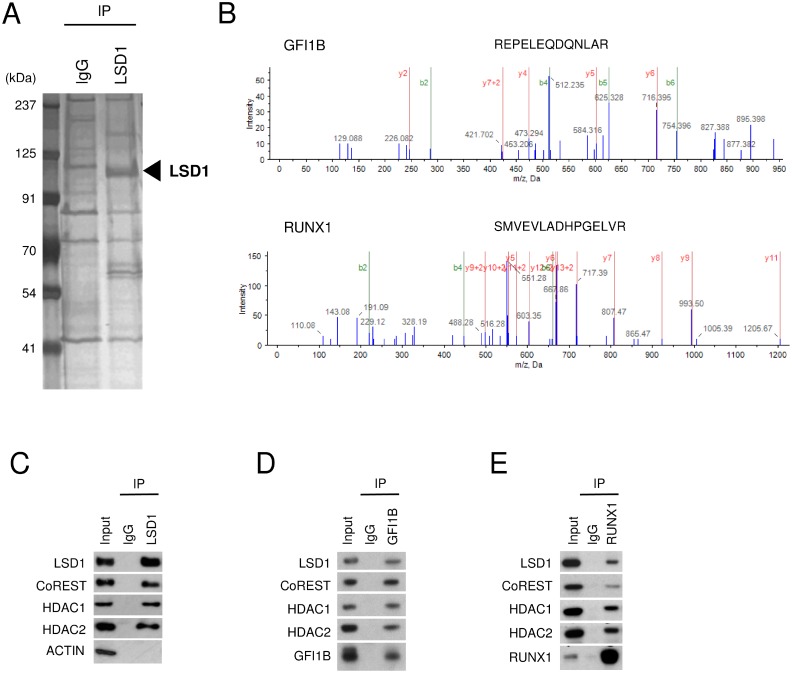
Interaction of LSD1 with GFI1B and RUNX1 in HEL cells (**A**) Silver staining of the LSD1-IP lysate. The IP lysate with normal IgG was used as a negative control. The left lane displays size markers. The arrow indicates the protein band corresponding to the size of LSD1. (**B**) Representative spectra of MS/MS show the identified sequence of the peptides derived from GFI1B and RUNX1 in the LSD1-IP lysate. (**C**–**E**) Co-IP-WB with anti-LSD1 (C), anti-GFI1B (D), or anti-RUNX1 antibody (E) in HEL cells. Normal IgG was used as a negative control. Indicated antibodies were used to detect target proteins. ACTIN was used as a negative control unbound to LSD1. Experiments in (C–E) were performed independently at least three times and the representative data are shown.

To identify proteins that are associated with LSD1 in HEL cells, we first purified the whole-cell lysate by co-immunoprecipitation (co-IP) with anti-LSD1 antibody (LSD1-IP). The LSD1-IP lysate showed not only a protein band corresponding to the size of LSD1 but also several protein bands which were not detected in the control IgG-IP lysate (Figure 1A). Liquid chromatography coupled with tandem mass spectrometry (LC-MS/MS) analyses successfully revealed a number of peptides in the LSD1-IP sample (Figure 1B). Finally, as LSD1 partners in HEL cells, we identified 54 proteins of which more than one peptide were detected with at least 95% confidence in the LSD1-IP sample but not in the control IgG-IP sample and which had reliable score calculated by the Pro Group algorithm [Unused ProtScore ≥ 2] (Table 1). They included components of BRAF-histone deacetylase complex (BHC) such as CoREST (also known as RCOR1), HDAC1, HDAC2, GSE1, HMG20A and HMG20B which were previously reported to form the LSD1-containing complex [11, 16]. In addition, key hematopoietic TFs such as RUNX1 and GFI1B were listed in the LSD1 partner list (Table 1).

**Table 1:** LSD1 partner candidates identified by LC-MS/MS in HEL cells

Rank	Symbol Name	UniProtKB number	Unused ProtScore	Peptides (95%)
1	GSE1	Q14687	101.90	55
2	KDM1A (LSD1)	O60341	89.31	62
3	HSPD1	P10809	71.24	48
4	RCOR1 (CoREST)	Q9UKL0	44.71	28
5	HDAC2	Q92769	31.70	23
6	HNRNPUL1	Q9BUJ2	21.59	11
7	THRAP3	Q9Y2W1	20.07	10
8	HMG20A	Q9NP66	19.73	11
9	SMARCA5	O60264	19.05	10
10	RCOR3	Q9P2K3	18.08	18
11	MYO1G	B0I1T2	16.31	9
12	BCLAF1	Q9NYF8	15.64	8
13	HDAC1	Q13547	15.01	21
14	RPS2	P15880	13.88	8
15	THBS1	P07996	13.77	7
16	HMG20B	Q9P0W2	13.44	7
17	SUPT16H	Q9Y5B9	12.01	6
18	UBTF	P17480	9.65	6
19	ERH	P84090	9.09	5
20	RPS4X	P62701	7.90	4
21	RPS13	P62277	6.18	3
22	RPL30	P62888	6.00	3
23	RUNX1	Q01196	5.96	3
24	NOP58	Q9Y2×3	5.78	4
25	TUFM	P49411	5.54	3
26	DKC1	O60832	5.27	4
27	HRNR	Q86YZ3	4.76	4
28	MDC1	Q14676	4.47	2
29	KHDRBS1	Q07666	4.36	2
30	SNU13	P55769	4.14	2
31	TFAM	Q00059	4.01	2
32	HNRNPD	Q14103	4.00	2
33	PPP1CA	P62136	4.00	2
34	ILF2	Q12905	4.00	2
35	RPA3	P35244	4.00	2
36	FABP5	Q01469	3.89	2
37	RPLP0	P05388	3.64	2
38	MYO1C	O00159	3.63	2
39	RPL28	P46779	3.61	3
40	RALY	Q9UKM9	3.54	2
41	RPL36	Q9Y3U8	3.48	2
42	SAFB2	Q14151	3.34	7
43	RPL32	P62910	3.24	2
44	ARPC4	P59998	3.09	2
45	SAP18	O00422	3.05	2
46	GFI1B	Q5VTD9	2.86	2
47	RPA1	P27694	2.67	2
48	PPP1R9B	Q96SB3	2.61	2
49	DDX21	Q9NR30	2.59	2
50	DSP	P15924	2.47	2
51	RPS24	P62847	2.45	2
52	PRDX1	Q06830	2.43	2
53	ILF3	Q12906	2.40	2
54	H3F3A	P84243	2.00	10
55	PHOX2B	Q99453	2.00	2

Only proteins with Peptides (95%) ≥ 2 and Unused ProtScore ≥ 2 are shown.

Unused ProtScore for a particular protein was calculated from the sum of all peptide evidence for that specific protein by the Pro Group algorithm.

Peptides (95%) means the number of distinct peptides having at least 95% confidence.

The LSD1-CoREST-HDAC1/2 complex was previously identified in various types of cells including neural cells, HEK293T cells, Hela cells, and blood cells [10, 11, 16, 17], and was reported to interact with GFI1B in murine blood cells [17]. To confirm the proper formation of the LSD1-CoREST-HDAC1/2 complex and the association of the complex with GFI1B and RUNX1 in HEL cells, we performed co-IP followed by western blotting (co-IP-WB). The formation of the LSD1-CoREST-HDAC1/2 complex was validated by co-IP-WB with anti-LSD1 antibody (Figure 1C). The interaction of the LSD1-CoREST-HDAC1/2 complex with GFI1B was also validated by co-IP-WB with anti-GFI1B antibody (Figure 1D). Furthermore, co-IP-WB with anti-RUNX1 antibody showed that RUNX1 interacts with the LSD1-CoREST-HDAC1/2 complex (Figure 1E). These data suggest that GFI1B and RUNX1 form a complex with LSD1, CoREST and HDAC 1/2 in HEL cells.

### Selective dissociation of GFI1B from the LSD1 complex by NCD38

**Figure 2:**
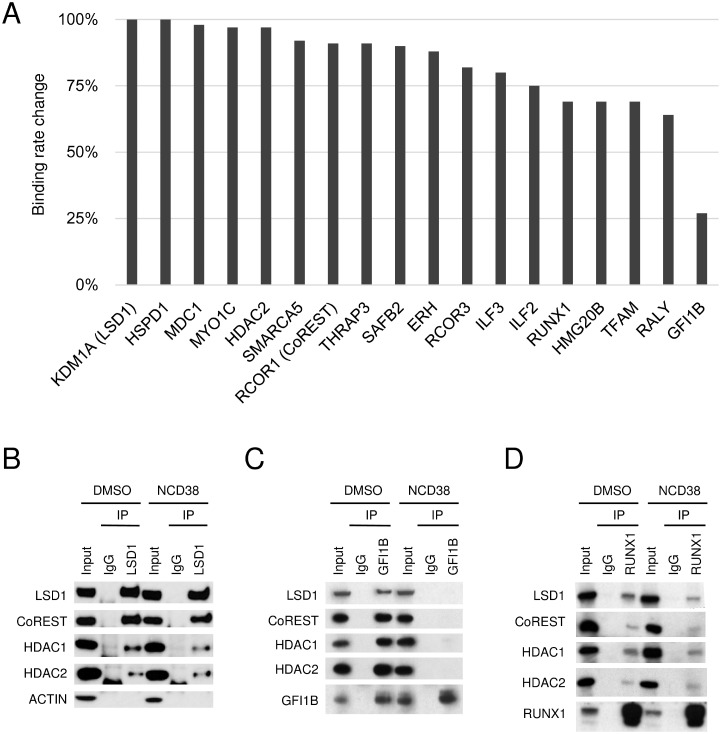
Selective impairment of the interaction between LSD1 and GFI1B by NCD38 (**A**) Binding rate change of LSD1 partners after the NCD38 treatment. The binding rate change was calculated as the normalized abundances in NCD38-treated cells divided by those in DMSO-treated cells. Values normalized to the binding rate change of LSD1 are shown. (**B**–**D**) Co-IP-WB with anti-LSD1 (B), anti-GFI1B (C), or anti-RUNX1 antibody (D) in HEL cells treated with DMSO or NCD38 for 24 hours. Normal IgG was used as a negative control. Indicated antibodies were used to detect target proteins. ACTIN was used as a negative control unbound to LSD1. Experiments in (B–D) were performed independently at least three times and the representative data are shown.

We previously reported that NCD38 exerts anti-leukemic effect against HEL cells through transdifferentiation from erythroid lineage to granulomonocytic lineage [15]. The other recent study also reported that another LSD1 inhibitor T-3775440 can cause the similar effect and disrupt the interaction between LSD1 and GFI1B in HEL cells [18]. Thus, we comprehensively investigated whether the interaction of LSD1 with the identified partners is altered by NCD38 treatment in HEL cells. The relative abundances of LSD1-interacting proteins in the presence or absence of NCD38 were estimated by label-free quantification (Supplementary Table 1). There were no key hematopoietic regulators which gained association with LSD1 in the presence of NCD38 by more than 1.5-folds. In contrast, the binding rate of GFI1B to LSD1 was markedly reduced to 27% after the NCD38 treatment. Of note, GFI1B showed the highest reduction in the binding rate to LSD1 while no other proteins exhibited such high reduction rate (Figure 2A). In fact, the binding rate of RUNX1 to LSD1 remained at a decrease to 69% and the binding of CoREST, HDAC1, and HDAC2 to LSD1 were retained irrespective of the NCD38 treatment. To validate the binding alterations of these proteins estimated by the mass spectrometry, we next performed co-IP-WB in the presence or absence of NCD38. Co-IP-WB with anti-LSD1 antibody revealed no impairment of the LSD1-CoREST-HDAC1/2 complex by NCD38 (Figure 2B). In clear contrast, co-IP-WB with anti-GFI1B antibody showed that NCD38 almost completely impaired the interaction of GFI1B with LSD1, CoREST, HDAC1 and HDAC2, supporting the data obtained from the mass spectrometry (Figure 2C). Co-IP-WB with anti-RUNX1 antibody showed that the interaction of RUNX1 with LSD1, CoREST, HDAC1, and HDAC2 was less impaired by NCD38 (Figure 2D). Altogether, these data suggest that a pharmacological action of NCD38 is a selective disruption of the interaction between GFI1B and the LSD1-CoREST-HDAC1/2 complex.

### Inverse correlation between *ERG* and *GFI1B* transcripts

**Figure 3:**
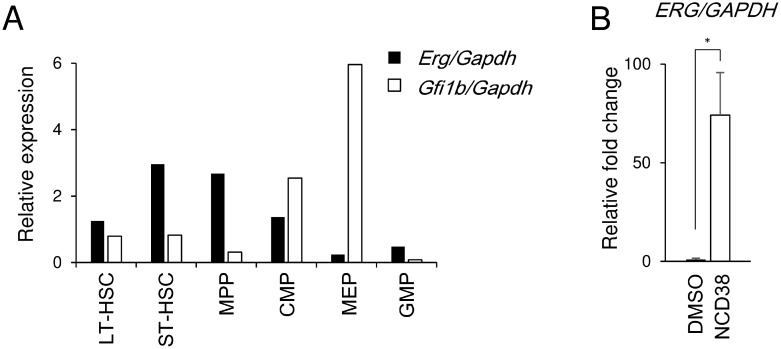
Inverse correlation between Erg and Gfi1b in MEP cells and de-repression of ERG by NCD38 in HEL cells (**A**) Relative expression of the *Erg* and *Gfi1b* transcripts of each hematopoietic fractions isolated from murine bone marrow. The data were normalized to the *Gapdh* transcript level. Experiments were performed independently twice and the means are displayed. LT-HSC, long-term hematopoietic stem cell; ST-HSC, short-term hematopoietic stem cell; MPP, multipotent progenitor; CMP, common myeloid progenitor; MEP, megakaryocyte-erythroid progenitor; GMP, granulocyte-monocyte progenitor. Sorting gates are shown in Supplementary Figure 1. (**B**) Relative fold change of the ERG transcript in HEL cells after treatment with NCD38 for 24 hours. The data are shown as the relative fold change in comparison to DMSO-treated HEL after normalization to GAPDH. The data are presented as mean with standard deviations for 3 independent experiments. Statistical comparison was performed using two-tailed Student *t* test. ^*^P < 0.01.

*ERG* was previously identified as one of the LSD1 target genes in HEL and other cell lines of acute myeloid leukemia and myelodysplastic syndromes [15]. ERG and GFI1B are known to be required for normal hematopoiesis [19, 20] and for erythroid maturation [21] respectively. Thus, we investigated the correlation between *Erg* and *Gfi1b* transcripts in developmental stages of murine hematopoiesis [22] (Supplementary Figure 1). The *Erg* transcript level was high in short-term hematopoietic stem cells (ST-HSCs) and multipotent progenitors (MPPs) but relatively decreased in common myeloid progenitors (CMPs) and was much lower in megakaryocyte-erythroid progenitors (MEPs) which are in the primitive stage of erythroid lineage (Figure 3A). In contrast, the *Gfi1b* transcript level relatively increased in CMPs and was much higher in MEPs in accordance with a previous report [23]. Furthermore, the *ERG* transcript was hardly detected in the basal state while that was induced after the NCD38 treatment in HEL cells (Figure 3B). These data suggest that the expression of ERG and GFI1B seems to be inversely correlated in hematopoiesis and give rise to the possibility that ERG might be suppressed by GFI1B in coordination with LSD1 in immature erythroid-lineage cells.

### Downregulation of an erythroid marker CD235a by ERG overexpression

**Figure 4:**
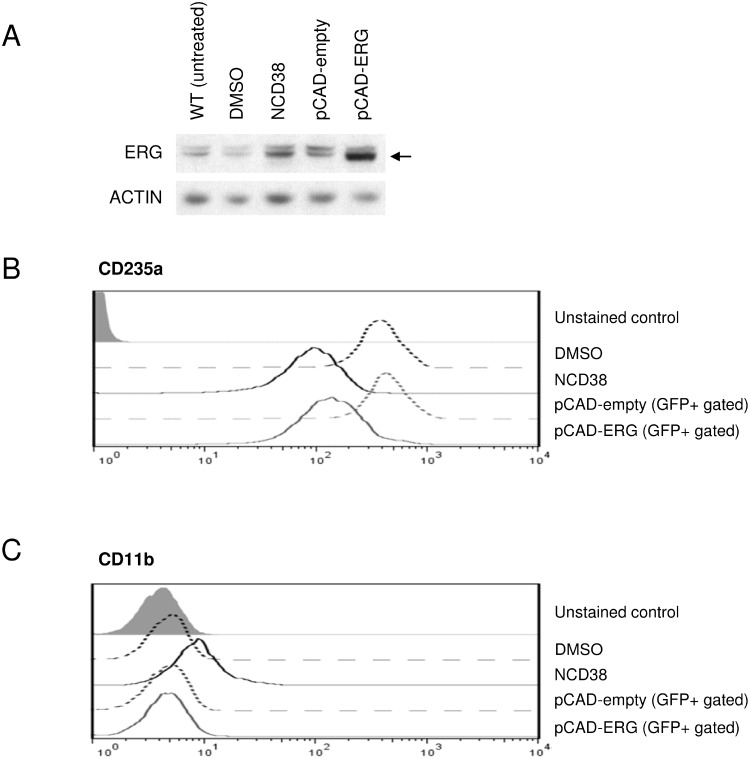
Lentiviral ERG overexpression mimics downregulation of the erythroid marker by NCD38 (**A**) ERG induction by NCD38 and overexpression by lentiviral transduction. Western blotting shows the ERG protein level (indicated by the arrow) in wild-type (WT, untreated), DMSO-treated, NCD38-treated, pCAD-empty-transduced, and pCAD-ERG-transduced HEL cells. Drug treatment time was 48 hours. ACTIN was used as an internal control. The schema of lentiviral vectors is shown in Supplementary Figure 2. (**B**–**C**) FACS analyses of CD235a (B) and CD11b (C). Histogram plots display CD235a or CD11b expression level on the cell surface of HEL cells treated with DMSO (black dotted line) or NCD38 (black solid line) for 48 hours, and of GFP-positive (GFP+ gated) HEL cells 3 days after transduction with pCAD-empty (gray dotted line) or pCAD-ERG (gray solid line). The gray filled histogram plots indicate unstained controls. The experiments were performed independently twice and the representative data are shown.

We next investigated whether upregulation of ERG could be responsible for the transdifferentiation of HEL cells induced by NCD38. Using the lentiviral transduction system, we successfully overexpressed ERG at the protein level comparable to that induced by NCD38 (Figure 4A, Supplementary Figure 2). NCD38 downregulated an erythroid lineage marker, CD235a (Figure 4B), and upregulated a myeloid lineage marker, CD11b (Figure 4C). On the other hand, lentiviral ERG overexpression caused comparable downregulation of CD235a (Figure 4B) but no change of CD11b (Figure 4C). These results clearly demonstrate that ERG overexpression attenuates the erythroid-lineage phenotype of HEL cells, suggesting that upregulation of ERG seems to contribute at least in part to the transdifferentiation by NCD38.

### Conservation of the GFI1B motif in the *ERG*-SE

**Figure 5:**
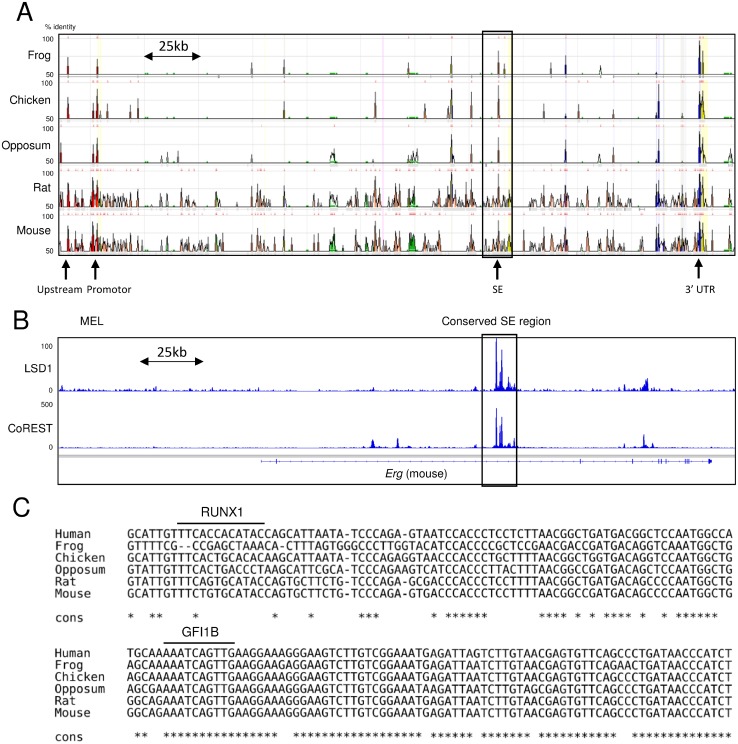
Occupation of LSD1 and conservation of the GFI1B binding motif in the *ERG-SE* (**A**) The view of the highly conserved regions at the *ERG* locus between human and the indicated species from ECR browser. The squared area is corresponding to the human *ERG-SE*. The height of the peaks indicates the degree of conservations between human and the indicated species. The arrows indicate highly conserved non-coding regions. (**B**) Publicly available ChIP-seq profiles of LSD1 and CoREST at the *Erg* locus in MEL cells. ChIP-seq data were visualized with IGV software. The squared area indicates the conserved SE region. (**C**) Alignment of the genomic sequences at the *ERG-SE* between the indicated species. The lines above the sequences indicate the binding motifs of RUNX1 or GFI1B. The asterisks indicate parts of the conserved sequences across the presented species.

In our previous study, chromatin immunoprecipitation coupled with massively parallel sequencing (ChIP-seq) analysis for H3K27ac revealed that NCD38 activates approximately 500 SEs, among which the *ERG*-SE is one of the highest ranked SEs according to the ROSE analysis [15]. Therefore, the pronounced increase of the *ERG* transcript by NCD38 is assumed to be caused by cancellation of suppression of the *ERG*-SE by LSD1. Because the *ERG*-SE area is highly conserved across vertebrates (Figure 5A), we surveyed the presence of LSD1 in the murine genomic region corresponding to the human *ERG*-SE using publicly available ChIP-seq data [24–26]. Analysis of ChIP-seq data from murine erythroleukemia (MEL) cells showed that both LSD1 and CoREST occupy the conserved SE region at the *Erg* gene. (Figure 5B). Moreover, the TF motif analysis revealed that the human *ERG*-SE contains the binding motifs of GFI1B and RUNX1, and that the binding motif of GFI1B but not RUNX1 is conserved across vertebrates (Figure 5C). These *in silico* findings suggest the possibility that LSD1 could suppress the *ERG*-SE by forming the complex with GFI1B in erythroleukemia cells.

### Reactivation of the *ERG*-SE by separating LSD1 and CoREST from GFI1B by NCD38

**Figure 6:**
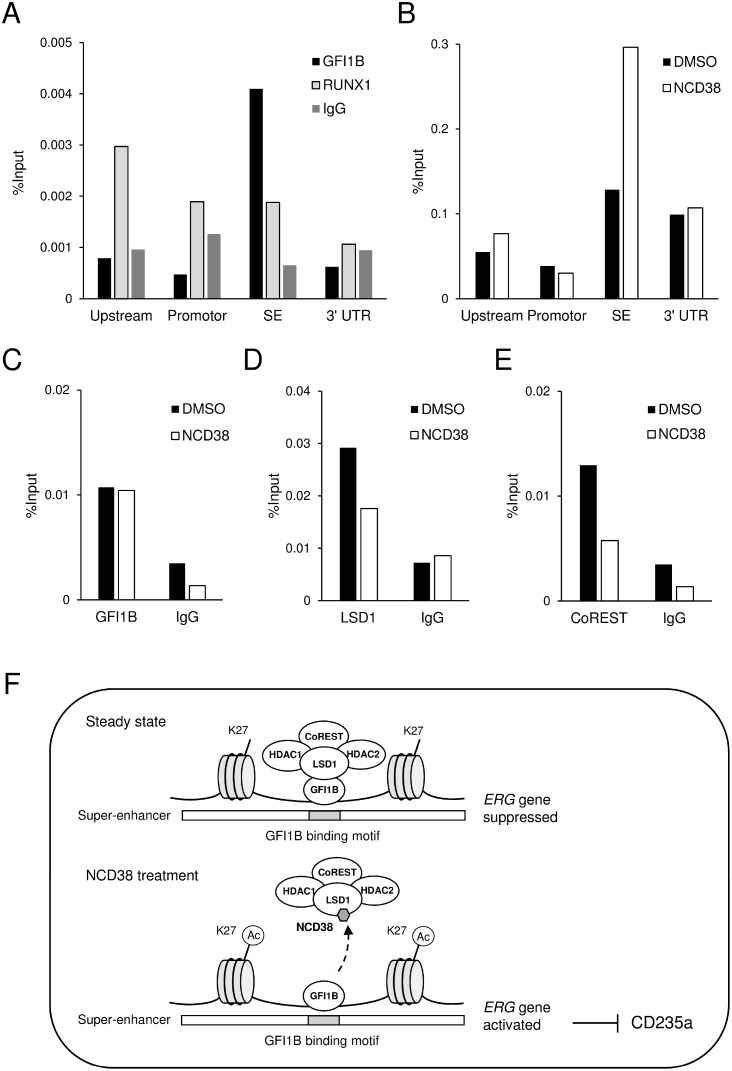
The *ERG-SE* is occupied by GFI1B and activated by the NCD38 treatment (**A**) ChIP-qPCR analysis for GFI1B, RUNX1, or control IgG in the conserved non-coding regions at the *ERG* locus in HEL cells. (**B**) ChIP-qPCR analysis for H3K27ac in the conserved non-coding regions at the *ERG* locus in HEL cells treated with DMSO or NCD38 for 24 hours. Each conserved non-coding region is indicated in Figure 5A. (**C**–**E**) ChIP-qPCR analysis for GFI1B (C), LSD1 (D), and CoREST (E) at the *ERG-SE* locus in HEL cells treated with DMSO or NCD38 for 24 hours. All ChIP-qPCR assays were performed independently twice and the means are displayed. (**F**) Illustration of the interaction of GFI1B and the LSD1-CoREST-HDAC1/2 complex at the *ERG-SE* in HEL cells. In the steady state, GFI1B binds to the conserved binding motif and represses the *ERG-SE* through recruiting the LSD1-CoREST-HDAC1/2 complex. NCD38 separates the complex from GFI1B to restore the activity of the *ERG-SE*.

To clarify the above possibility, we first investigated whether GFI1B specifically occupies the *ERG*-SE in HEL cells. Chromatin-immunoprecipitation followed by quantitative PCR (ChIP-qPCR) analysis for GFI1B and RUNX1 was performed at highly conserved non-coding areas including upstream, promotor, 3’ UTR, and SE regions (indicated in Figure 5A). As a result, GFI1B highly condensed at the SE region compared to other regions, while RUNX1 mainly condensed at the upstream region (Figure 6A). Furthermore, ChIP-qPCR analysis for H3K27ac revealed that the level of H3K27ac was elevated only at the SE region after the NCD38 treatment (Figure 6B). These results indicate that GFI1B but not RUNX1 specifically binds to the *ERG*-SE and could be involved in suppression of the *ERG*-SE. We next investigated how NCD38 alters the binding status of GFI1B, LSD1, and CoREST to the *ERG*-SE. ChIP-qPCR analyses at the SE region showed that the occupancy level of GFI1B was not changed (Figure 6C) but that of LSD1 and CoREST was reduced by NCD38 (Figure 6D, 6E). These results indicate that the binding of GFI1B to the *ERG*-SE is retained while LSD1 and CoREST are dissociated from the *ERG*-SE by NCD38. Given the selective disruption of the interaction between GFI1B and the LSD1-CoREST-HDAC1/2 complex by NCD38, these data collectively suggest that GFI1B represses the *ERG*-SE through recruiting the complex and that NCD38 restores the activity of the *ERG*-SE by separating the complex from GFI1B (Figure 6F).

## DISCUSSION

The comprehensive proteome analysis identified GFI1B and RUNX1 as major LSD1 partners that can recruit LSD1 on specific genomic regions in HEL cells. Other key hematopoietic TFs were not listed up as significant LSD1 partners at least in our result. GFI1B has been reported to interact with the LSD1-CoREST-HDAC1/2 complex via the SNAG repression domain and suppress myeloid differentiation [17]. RUNX1 has been reported to interact with LSD1 and CoREST to repress hematopoietic genes in differentiated MEL cells [27]. Therefore, GFI1B and RUNX1 presumably play crucial roles in erythroid-lineage differentiation or maintenance by utilizing LSD1. However, it is unclear how LSD1 selectively interacts with these two TFs in HEL cells, even though another study reported that LSD1 is associated with the T cell leukemia oncoprotein (TAL1/SCL) [28], which functions essentially in the early development of hematopoiesis [29]. This selectivity of LSD1 to GFI1B and RUNX1 is required to be investigated in the future.

Several LSD1 inhibitors including NCD38 are promising as anti-leukemic agents while the detailed mechanisms are less known [15, 30, 31]. Our previous report demonstrated that NCD38 induces transdifferentiation from erythroid lineage to granulomonocytic lineage with de-repression of approximately 500 SEs in HEL cells [15]. Therefore, in the current study, we attempted to comprehensively understand the relationship between LSD1 and TFs on SEs using the *ERG*-SE as an example. The comprehensive proteome comparison between the presence and absence of NCD38 revealed that NCD38 selectively disrupts the interaction between LSD1 and GFI1B. Furthermore, the ChIP-qPCR analyses clearly showed that GFI1B specifically occupies the *ERG*-SE and that LSD1 and CoREST but not GFI1B are dissociated from the *ERG*-SE by NCD38. These data indicate that the selective dissociation of LSD1 from GFI1B on the *ERG*-SE by NCD38 restores the *ERG*-SE activation inducing ERG expression. In addition, the lentiviral ERG overexpression fulfilled a part of the transdifferentiation by NCD38. Collectively, this selective dissociation could contribute, at least in part, to anti-leukemia effects. It was an unexpected effect of NCD38 because NCD38 was originally designed to target the center of enzymatic activity and actually exhibits the strong inhibitory activity towards LSD1 [32]. However, the mechanism that NCD38 exerts anti-leukemia effects was unlikely to depend only on its inhibitory activity because NCD38 could quickly elevate the H3K27ac level on specific SEs despite the absence of DNA-binding motifs and deacetylation activity in LSD1 [15]. The current study could provide a new aspect of NCD38 that makes it possible to explain these questions. Recently the specific protein-protein interactions (PPIs) have begun to be expected as novel drug targets [33]. The current findings suggest that the interaction between LSD1 and GFI1B is presumably an important PPI at least in erythroleukemia cells. In addition, it might be possible to classify NCD38 not only as a simple LSD1 inhibitor but also as a new PPI-targeting small molecule. According to the recent report, the interaction between LSD1 and GFI1B was also disrupted by another LSD1 inhibitor T-3775440 [18]. Although its selectivity of TFs and association with SEs were not mentioned, the basic structure of NCD38 and T-3775440 are very similar, implying that these drugs might act on leukemia cells through the same pharmacological mechanism. Structural analyses about how the GFI1B-LSD1 complex is altered and dissociated by these small molecules would be necessary to create more agile drugs which target the PPI between LSD1 and GFI1B.

GFI1B is highly expressed during erythroid and megakaryocytic maturation and suppresses non-erythroid specific genes [21]. Although several studies have reported that this suppression is regulated via the promoters [34, 35], our data propose that the repression of SEs by GFI1B and LSD1 may also play an important role in erythroid differentiation. The *ERG*-SE is identical to the *ERG* stem cell enhancer in previous reports [36, 37]. Several key hematopoietic TFs including SCL, LYL1, PU.1, LMO2, GATA2, RUNX1, FLI1 and ERG occupy and activate the *ERG*-SE leading to the upregulation of *ERG* expression in human CD34 positive hematopoietic stem and progenitor cells. In addition, according to ChIP-seq analyses conducted in murine blood cells by another group, the H3K27ac level at the *Erg*-SE is the highest in MPPs but hardly detected in MEPs [6]. Therefore, downregulation of Erg in MEPs that was shown in Figure 3A might be caused by the repression of the *Erg*-SE. On the other hand, it would be plausible to argue that this repression in MEPs might result from downregulation of PU.1 and Gata2 because they are known to be repressed in erythroid progenitors [1, 38]. However, we previously reported that NCD38 could activate the *ERG*-SE without altering the transcript level of PU.1 and GATA2 in HEL cells [15]. The current study does not clarify whether Erg is downregulated especially in MEPs by exactly the same mechanism as shown in this report, but gives rise to the possibility that LSD1 and TFs may cooperate to silence lineage-specific regulators by inactivating their SEs and to control the cell fate. Further investigation what TFs are associated with LSD1 in each lineage will be required to better understand the precise hematopoietic transcriptional networks.

## MATERIALS AND METHODS

### Cell culture and reagent

HEL cells were maintained in RPMI-1640 supplemented with 10% heat-inactivated fetal bovine serum (FBS). Lenti-X 293T cells (Takara, Kusatsu, Japan) were grown in Dulbecco Modified Eagle's Medium (DMEM) supplemented with 10% FBS. NCD38 was synthesized as previously described [32], dissolved in dimethyl sulfoxide (DMSO) and used at 2 μM for all experiments.

### Mice

C57BL/6 mice were purchased from Charles River Laboratories Japan (Yokohama, Japan) or CLEA Japan, Inc. (Tokyo, Japan), and maintained under specific pathogen-free conditions at the Centre for Experimental Animals of Kyoto University or at the Research Center for Animal Life Science of Shiga University of Medical Science. The animal experiments were approved by the animal research committee of Kyoto University or Shiga University of Medical Science and performed in accordance with the institutional guidelines.

### Antibodies

The antibodies for immunoprecipitation were anti-LSD1 (ab17721, Abcam, Cambridge, UK), anti-GFI1B (sc-28356X, Santa Cruz Biotech, Dallas, TX, USA), anti-RUNX1 (ab23980, Abcam), anti-H3K27ac (39133, Active Motif, Carlsbad, CA, USA), anti-CoREST (ab32631, Abcam), normal rabbit IgG (sc-2027, Santa Cruz), and normal mouse IgG (sc-2025, Santa Cruz). The primary antibodies for western blotting were anti-LSD1 (C69G12, Millipore, Billerica, MA, USA), anti-CoREST (ab32631, Abcam), anti-HDAC1 (ab7028, Abcam), anti-HDAC2 (ab7029, Abcam), anti-GFI1B (sc-28356X or sc-22795, Santa Cruz), anti-RUNX1 (ab23980, Abcam, or sc-365644, Santa Cruz), anti-ERG (ab133264, Abcam), and anti-ACTIN (sc-1616, Santa Cruz). The secondary antibodies were anti-rabbit IgG (NA934V, GE Healthcare, Little Chalfont, UK), anti-mouse IgG (NA931V, GE Healthcare), and anti-goat IgG (sc-2020, Santa Cruz) conjugated with horseradish peroxidase.

### Co-immunoprecipitation assay

For co-IP assays, 10^7^ cells were washed twice by PBS, lysed in N450 buffer (50 mM Tris-HCl pH 7.5, 450 mM NaCl, 1% NP-40, 5 mM EDTA pH 8.0, 1 mM MgCl_2_, 5% Glycerol) and agitated for 30 min at 4° C. After centrifugation, the supernatants were mixed with 2 volumes of N0 buffer (50 mM Tris-HCl pH 7.5, 1% NP-40, 5 mM EDTA pH 8.0, 1 mM MgCl_2_, 5% Glycerol). After preclear by incubation with 10 μL of Dynabeads Protein A or G (Thermo Fisher Scientific, Waltham, MA, USA) for 30 min at 4° C, the supernatants were incubated with 1–2 μg of an indicated antibody for 1–3 hours at 4° C, followed by immunoprecipitation with 20 μL of the same beads for 3 hours at 4° C. For co-IP with anti-LSD1 antibody for mass spectrometry analyses or with anti-RUNX1 antibody, beads were conjugated with each antibody using the Dynabeads Antibody coupling kit (Thermo Fisher Scientific) according to the manufacturer's instruction and then used for 3-hour immunoprecipitation. The beads were washed five times with N150 buffer (50 mM Tris-HCl pH 7.5, 150 mM NaCl, 1 % NP-40, 5 mM EDTA pH 8.0, 1 mM MgCl_2_, 5% Glycerol) and then boiled for 5 min after adding sample buffer. For co-IP using antibody-conjugated beads, the beads were incubated with elution buffer (0.5 M NH_4_OH, 0.5 mM EDTA pH 8.0) for 5 min at room temperature. The protease inhibitor cocktails (Nacalai Tesque, Kyoto, Japan) were added to N450, N0, and N150 buffers just before use. Silver staining was performed by using the Silver Staining MS kit (Wako, Osaka, Japan) according to the manufacturer's instruction.

### In-solution protein digestion

The eluates from the immunoprecipitation beads were precipitated with cold acetone and resuspended in 8 M Urea/30 mM ammonium bicarbonate. The resuspension was subjected to the reductive alkylation with dithiothreitol and iodoacetamide. One μg of tosyl phenylalanyl chloromethyl ketone-treated trypsin (Thermo Fisher Scientific) was added to the solution and the proteins were digested for overnight. The digestion products were purified using C-18 spin column (Thermo Fisher Scientific) according to the manufacturers’ instruction, and resuspended in 0.1% formic acid before subjection to the mass spectrometry.

### Mass spectrometry

The protein digests were separated using Nano-LC-Ultra 2D-plus equipped with cHiPLC Nanoflex (Eksigent, Dublin, CA, USA) in trap-and-elute mode, with trap column (200 μm × 0.5 mm ChromXP C18-CL 3 μm 120 Å (Eksigent)) and analytical column (75 μm × 15 cm ChromXP C18-CL 3 μm 120 Å (Eksigent)). The separation was carried out with a binary gradient with solvent A (98% water, 2% acetonitrile, 0.1% formic acid) and solvent B (20% water, 80% acetonitrile, 0.1% formic acid). The gradient program was 2 to 40% B for 250 min, 40 to 90% B in 1 min, 90% B for 5 min, 90 to 2% B in 0.1 min, and 2% B for 18.9 min, at 300 nL/min. The eluates were infused on-line to a mass spectrometer (TripleTOF 5600 + System with NanoSpray III source and heated interface (SCIEX, Framingham, MA, USA)) and ionized in an electrospray ionization-positive mode. Data acquisition was carried out with an information-dependent acquisition method. The acquired datasets were analyzed by ProteinPilot software version 4.5beta (SCIEX) with the UniProtKB/Swiss-Prot database for human (June 2014) appended with known common contaminants (SCIEX). The quality of the database search was confirmed by the false discovery rate analysis in which the reversed amino acid sequences were used as decoy. The protein identifications were evaluated by the numbers of identified peptides with at least 95% confidence, and the corresponding Unused ProtScores that were calculated by the Pro Group algorithm (SCIEX).

### Label-free quantification of the relative protein abundance

The LC-MS/MS datasets acquired by TripleTOF 5600 + System were imported to the platform of Progenesis QI for proteomics software (Nonlinear Dynamics, Newcastle upon Tyne, UK) for the relative quantification [39]. The relative abundance of each peptide was calculated using normalize to all proteins method. The identification of peptides was carried out by importing the corresponding peptide identification results generated by ProteinPilot software. The relative abundance of each protein was calculated by the grouping non-conflicting peptides method.

### Real-time quantitative PCR

RNA extractions were prepared using TRIzol reagent (Thermo Fisher Scientific) or the RNeasy Micro Kit (Qiagen, Hilden, Germany), and cDNAs were synthesized by Superscript II reverse transcriptase and Oligo (dT) primers (Thermo Fisher Scientific). Quantitative PCR (qPCR) of mRNA and ChIP-DNA were performed using Thunderbird SYBR qPCR Mix (Toyobo, Osaka, Japan) and TaKaRa Dice Real-Time TP800 system (Takara), or LightCycler 2.0 instrument (Roche Applied Science, Mannheim, Germany). Primer sequences are shown in Supplementary Table 2.

### Flow cytometry

For sorting murine hematopoietic stem and progenitor cells, FITC-conjugated anti-CD34 (RAM34), PE-conjugated anti-FcgRII/III (93), pacific blue-conjugated anti-Sca1 (D7), APC-conjugated anti-c-Kit (2B8), and biotin-conjugated anti-CD135 (A2F10) antibodies followed by APC-Cy7-conjugated streptavidin were used. For excluding lineage positive cells, PE-Cy5-conjugated anti-CD3ε (145–2C11), CD4 (GK1.5), CD8a (53–6.7), CD19 (eBio1D3), B220 (RA3-6B2), Gr-1 (RB6-8C5), TER-119 (TER-119), and CD11b (M1/70) antibodies were used. Cell sorting experiments were performed using a FACS Aria flow cytometer (BD Biosciences, San Jose, CA, USA). For flow cytometric analysis of HEL cells, PE-Cy5-conjugated anti-human CD11b (ICRF44) and CD235a (GA-R2) antibodies were used. Flow cytometric analyses were performed using a FACS Calibur (BD Biosciences). These antibodies were purchased from eBioscience (San Diego, CA, USA), BD Biosciences, or Biolegend (San Diego, CA, USA).

### Chromatin immunoprecipitation (ChIP)

ChIP assays for GFI1B, RUNX1, H3K27ac, LSD1 and CoREST were performed as described previously [15]. Briefly, after crosslinking with 0.5% or 1% paraformaldehyde for 10 min and quenching with 100 mM glycine for 10 min, cells were lysed and incubated in lysis buffer (50 mM HEPES pH 7.9, 140 mM NaCl, 1 mM EDTA pH 8.0, 10% Glycerol, 0.5% NP-40, 0.25% TritonX-100) on ice for 10 min. After two washes, the pellets were resuspended in shearing buffer (0.1% SDS, 1 mM EDTA pH 8.0, 10 mM Tris-HCl pH 8.0) and sonicated using S220 ultrasonicators (Covaris, Woburn, MA, USA). The sonicated samples were incubated overnight at 4° C with 2 or 4 μg of an indicated antibody and precipitated with 20 μL of Dynabeads Protein A or protein G. After extensive wash, the precipitated magnetic beads were treated with elution buffer (50 mM Tris-HCl pH 8.0, 10 mM EDTA pH 8.0, 1% SDS). Cross-links of the eluted samples were reversed by incubation for overnight at 65° C. After digestion of RNA and protein with RNase A and Proteinase K, DNA was finally purified using a Min Elute PCR Purification Kit (Qiagen) and analyzed by real-time qPCR.

### Lentiviral transduction

For *ERG* overexpression, the pCMV-Sport6 vector encoding the full-sequence human *ERG* gene (Clone ID: 6052140, GenBank™ accession number: BC040168) was purchased from Dharmacon (Lafayette, CO, USA) and the *ERG* coding part was moved to pCAD lentiviral vector equipped with IRES-GFP [4]. Lentiviral supernatants were generated in Lenti-X 293T cells as previously described [4], and infected to HEL cells for 24 hours. After 3 days, transduced HEL cells were analyzed by FACS. After 5 days, GFP positive cells were sorted using a FACS Aria flow cytometer (BD Biosciences), lysed in RIPA buffer (10 mM Tris-HCl pH 7.5, 150 mM NaCl, 5 mM EDTA pH 8.0, 1% Triton X-100, 1% sodium deoxycholate, 0.1% SDS, 1 mM PMSF) with the protease inhibitor cocktail, and analyzed by western blotting.

### ChIP-seq analysis, identification of conserved genomic regions, and TF motif analysis

The publicly available ChIP-seq data obtained from Gene Expression Omnibus database; LSD1 (Series GSE59859; Sample GSM1448833) [24], and CoREST (Series GSE36030; Sample GSM1003789) [25, 26] were visualized with the Integrative Genomics Viewer (IGV) software version 2.3 (Broad Institute, Cambridge, MA, USA; https://www.broadinstitute.org/igv). Genomic regions conserved among the species were identified using ECR browser (http://ecrbrowser.dcode.org/). Genomic sequence spanning the *ERG* locus was obtained from UCSC genome browser (https://genome.ucsc.edu/). DNA sequence alignments were performed using T-coffee server (http://tcoffee.vital-it.ch/apps/tcoffee/do:regular). TF binding sites were analyzed with R-VISTA (http://rvista.dcode.org/).

### Statistics

Statistical comparison was performed using two-tailed Student *t* test.

## SUPPLEMENTARY MATERIALS FIGURES AND TABLES




