# Supplementary material for: Selective dissociation between LSD1 and GFI1B by a LSD1 inhibitor NCD38 induces the activation of ERG super-enhancer in erythroleukemia cells
Source: Oncotarget. 2018 Apr 20;9(30):21007–21. doi: 10.18632/oncotarget.24774 (PMC5940392; doi:10.18632/oncotarget.24774)
Supplement: Supplementary file 1 [file oncotarget-09-21007-s001.pdf]

Selective dissociation between LSD1 and GFI1B by a LSD1 inhibitor NCD38 induces the activation of *ERG* super-enhancer in erythroleukemia cells

SUPPLEMENTARY MATERIALS

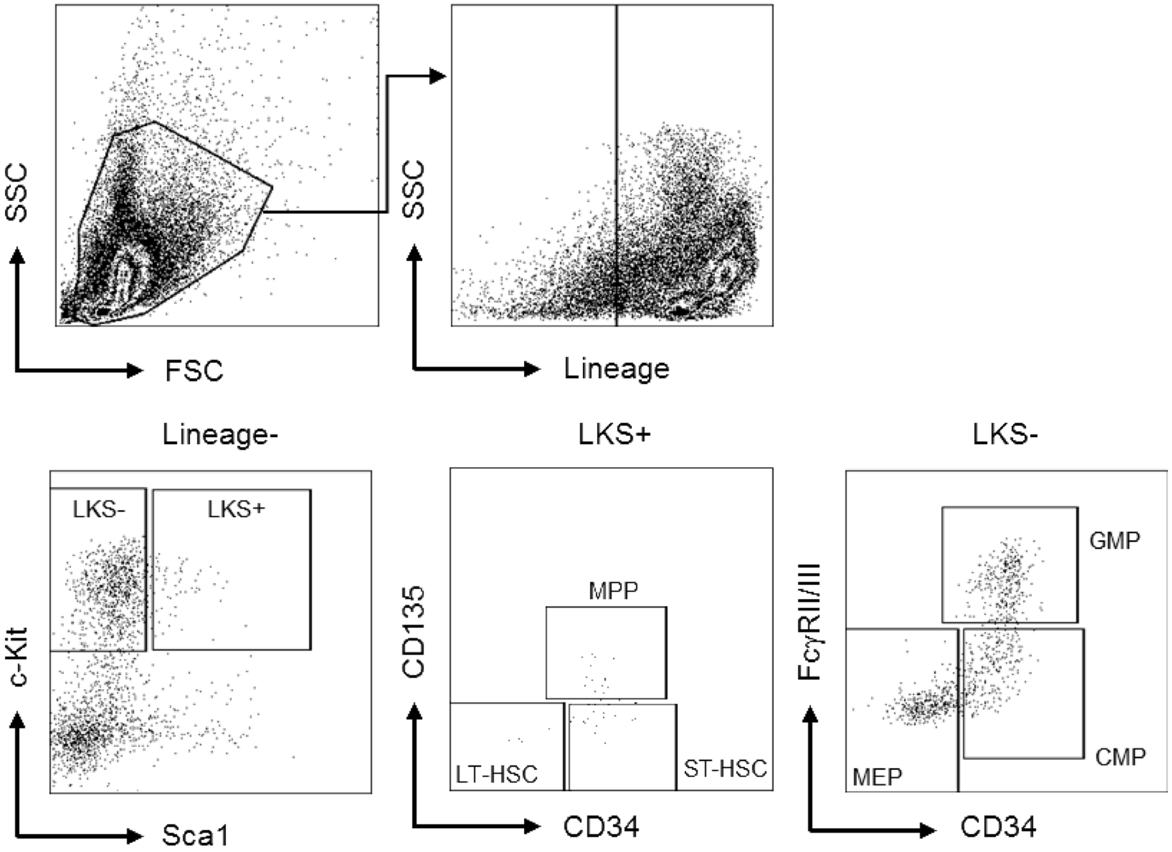

Supplementary Figure 1:FACS plots and sorted gates of murine hematopoietic stem and progenitor cells from bone marrow. Lineage includes CD3ε CD4, CD8a, CD19, B220, Gr-1, TER-119, and CD11b. FSC, forward scatter; SSC, side scatter; LT-HSC, long-term hematopoietic stem cell (Lineage$^-$Kit$^+$Sca1$^+$CD135$^-$CD34$^-$); ST-HSC, short-term hematopoietic stem cell (Lineage$^-$Kit$^+$Sca1$^+$CD135$^-$CD34$^+$); MPP, multi-potent progenitor (Lineage$^-$Kit$^+$Sca1$^+$CD135$^+$CD34$^+$); CMP, common myeloid progenitor (Lineage$^-$Kit$^+$Sca1$^-$CD34$^+$FcγRII/III^{mid}); GMP, granulocyte-macrophage progenitor (Lineage$^-$Kit$^+$Sca1$^-$CD34$^+$FcγRII/III^{high}); MEP, megakaryocyte-erythroid progenitor (Lineage$^-$Kit$^+$Sca1$^-$CD34$^-$FcγRII/III^{low}).

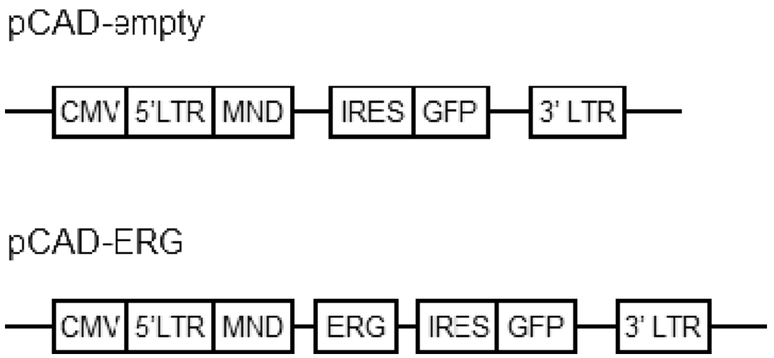

Supplementary Figure 2: Schematic representation of pCAD lentiviral vectors. The ERG part was inserted just before the IRES-GFP part. pCAD-empty was used as a control.

Supplementary Table 1: Relative protein levels determined by the label-free quantification in anti-LSD1-IPed lysates from HEL cells treated with DMSO or NCD38 for 24 hours. See Supplementary _Table_1

Supplementary Table 2: Primer sequences for RT-qPCR and ChIP-qPCR assay

**Primers for RT-qPCR**		
murine *Erg*	Forward	GGAGTGCAACCCTAGTCAGG
	Reverse	TAGCTGCCGTAGCTCATCC
murine *Gfi1b*	Forward	CTTACCACTGTGTCAAGTGCAAC
	Reverse	CTCCTGTGAGTGGACGTGAGTAT
murine *Gapdh*	Forward	AGGTCGGTGTGTGAACGGATTTG
	Reverse	TGTAGACCATGTAGTTGAGGTCA
human *ERG*	Forward	GCTGCTCAACCATCTCCTTC
	Reverse	ACAGGAGCTCCAGGAGGAAC
human *GAPDH*	Forward	GAAGGTGAAGGTCGGAGTC
	Reverse	GAAGATGGTGATGGGATTTC

Primers for ChIP-qPCR		
ERG Upstream	Forward	ATGTACACACCGCGTCTCTC
	Reverse	GGAGGATGAAAGGAATACCTATGC
ERG Promotor	Forward	CTGATTGTGAGGGAGGAGTG
	Reverse	TGGAATGTCTGGGAGCTAAG
ERG SE	Forward	CCACTCCGCATTGTTTCACC
	Reverse	CAGGGCTGAACACTCGTTAC
ERG 3′UTR	Forward	GCACAAGTTCCTGGACAAAG
	Reverse	GGCCTAGCATGGCAAATCAG
