# Supplementary material for: Selective dissociation between LSD1 and GFI1B by a LSD1 inhibitor NCD38 induces the activation of ERG super-enhancer in erythroleukemia cells
Source: Oncotarget. 2018 Apr 20;9(30):21007–21. doi: 10.18632/oncotarget.24774 (PMC5940392; doi:10.18632/oncotarget.24774)
Supplement: Supplementary file 2 [file oncotarget-09-21007-s002.pdf]

Supplementary Table 1: Relative protein levels determined by the label-free quantification in anti-LSD1-IPed lysates from HEL cells treated with DMSO or NCD38 for 24 hours

Rank	Symbol Name	Normalized abundance		Binding rate change (relative to LSD1)
		DMSO	NCD38	
1	GFI1B	2450.74	578.38	**0.27**
2	RALY	9190.93	5203.07	**0.64**
3	RUNX1	20286.69	12317.74	**0.69**
4	HMG20B	8244.04	5044.29	**0.69**
5	TFAM	4998.11	3073.01	**0.69**
6	ILF2	5043.33	3351.03	**0.75**
7	ILF3	16773.42	11929.33	**0.80**
8	RCOR3	15997.04	11606.13	**0.82**
9	ERH	18057.56	14152.87	**0.88**
10	SAFB2	12662.42	10114.59	**0.90**
11	RCOR1 (CoREST)	105584.30	84764.60	**0.91**
12	THRAP3	7603.18	6138.81	**0.91**
13	SMARCA5	33831.86	27628.35	**0.92**
14	MYO1C	7466.18	6420.09	**0.97**
15	HDAC2	32434.35	27896.01	**0.97**
16	MDC1	30162.60	26264.13	**0.98**
17	HSPD1	143068.53	126557.00	**1.00**
18	KDM1A (LSD1)	251942.03	223251.95	**1.00**
19	PPP1CA	222.89	200.07	**1.01**
20	RPS24	4414.65	4004.47	**1.02**
21	HDAC1	15903.88	14524.94	**1.03**
22	GSE1	168752.96	159934.29	**1.07**
23	RPS13	5985.49	5854.89	**1.10**
24	RPS2	8886.75	8895.24	**1.13**
25	HNRNPUL1	53798.65	55470.44	**1.16**

26	MYO1G	10306.10	10699.08	**1.17**
27	SNU13	9593.77	10136.77	**1.19**
28	SUPT16H	15217.22	16745.17	**1.24**
29	HNRNPD	2366.44	2657.81	**1.27**
30	HMG20A	20592.35	23151.21	**1.27**
31	RPL30	5992.75	6964.79	**1.31**
32	RPA1	19553.49	22783.36	**1.31**
33	PPP1R9B	2554.48	2990.23	**1.32**
34	RPS4X	4457.03	5248.08	**1.33**
35	DKC1	14200.99	16867.94	**1.34**
36	H3F3A	29388.96	35304.15	**1.36**
37	KHDRBS1	11582.43	14021.89	**1.37**
38	NOP58	10517.74	12918.56	**1.39**
39	RPL32	3555.03	4601.06	**1.46**
40	RPL36	2034.55	2642.23	**1.47**
41	RPA3	7008.06	9201.67	**1.48**
42	PRDX1	2659.71	3574.59	**1.52**
43	THBS1	3041.90	4096.82	**1.52**
44	DDX21	8275.70	11240.09	**1.53**
45	TUFM	4411.84	6155.16	**1.57**
46	SAP18	1869.67	3129.15	**1.89**
47	UBTF	35876.04	60778.59	**1.91**
48	BCLAF1	11646.69	20205.09	**1.96**
49	RPL28	3819.49	7392.51	**2.18**
50	ARPC4	835.38	1635.10	**2.21**
51	DSP	67.69	374.51	**6.24**

Binding rate change was calculated as the normalized abundances in NCD38 divided by those in DMSO. Values normalized to the binding rate change of LSD1 are shown.
